# The role of re-resection in recurrent hepatocellular carcinoma

**DOI:** 10.1007/s00423-022-02545-1

**Published:** 2022-05-23

**Authors:** Jan Bednarsch, Zoltan Czigany, Lara R. Heij, Iakovos Amygdalos, Daniel Heise, Philip Bruners, Tom F. Ulmer, Ulf P. Neumann, Sven A. Lang

**Affiliations:** 1 Department of Surgery and Transplantation, University Hospital RWTH, Aachen, Pauwelsstrasse 30, 52074 Aachen, Germany; 2 Institute of Pathology, University Hospital RWTH, Aachen, Pauwelsstrasse 30, 52074 Aachen, Germany; 3 Department of Radiology, University Hospital RWTH, Aachen, Pauwelsstrasse 30, 52074 Aachen, Germany; 4 Department of Surgery, Maastricht University Medical Centre (MUMC), Maastricht, the Netherlands

**Keywords:** HCC, Tumor recurrence, Surgery, Re-resection

## Abstract

**Purpose:**

While liver resection is a well-established treatment for primary HCC, surgical treatment for recurrent HCC (rHCC) remains the topic of an ongoing debate. Thus, we investigated perioperative and long-term outcome in patients undergoing re-resection for rHCC in comparative analysis to patients with primary HCC treated by resection.

**Methods:**

A monocentric cohort of 212 patients undergoing curative-intent liver resection for HCC between 2010 and 2020 in a large German hepatobiliary center were eligible for analysis. Patients with primary HCC (*n* = 189) were compared to individuals with rHCC (*n* = 23) regarding perioperative results by statistical group comparisons and oncological outcome using Kaplan–Meier analysis.

**Results:**

Comparative analysis showed no statistical difference between the resection and re-resection group in terms of age (*p* = 0.204), gender (*p* = 0.180), ASA category (*p* = 0.346) as well as main preoperative tumor characteristics, liver function parameters, operative variables, and postoperative complications (*p* = 0.851). The perioperative morbidity (Clavien-Dindo ≥ 3a) and mortality were 21.7% (5/23) and 8.7% (2/23) in rHCC, while 25.4% (48/189) and 5.8% (11/189) in primary HCC, respectively (*p* = 0.851). The median overall survival (OS) and recurrence-free survival (RFS) in the resection group were 40 months and 26 months, while median OS and RFS were 41 months and 29 months in the re-resection group, respectively (*p* = 0.933; *p* = 0.607; log rank).

**Conclusion:**

Re-resection is technically feasible and safe in patients with rHCC. Further, comparative analysis displayed similar oncological outcome in patients with primary and rHCC treated by liver resection. Re-resection should therefore be considered in European patients diagnosed with rHCC.

**Supplementary Information:**

The online version contains supplementary material available at 10.1007/s00423-022-02545-1.

## Introduction

Hepatocellular carcinoma (HCC) is a significant health-burden worldwide and the third most common cause of cancer-related mortality from a global perspective [1, 2]. Liver resection (LR) is the gold-standard treatment for patients with early HCC and nowadays even considered in individuals with advanced tumor stages [3–5]. Especially in patients with limited disease and preserved liver function, 10-year survival rates above 50% have been reported in selected cohorts [6]. However, as HCC arises on the background of chronic liver disease, tumor recurrence in the remnant liver, even after R0 resection, is reported in up to 80% of patients [7]. Salvage liver transplantation (SLT) might be an appropriate option for patients with recurrent HCC (rHCC) since it addresses both, the underlying liver damage and the oncological disease [8]. Unfortunately, a significant proportion of HCC patients do not qualify for transplantation due to older age, advanced tumor stages as well as major comorbidities or presence of other contraindications such as active alcohol abuse [9]. Moreover, the scarcity of liver grafts from deceased donors results in strict allocation rules limiting the utilization of SLT in this setting [8].

LR as therapy of primary HCC is well established and supported by a number of international guidelines. In contrast, the role of LR in rHCC remains to be determined [7, 10]. Especially for western patients, the evidence on LR in rHCC is limited, since most of the larger monocentric series are from Asian centers [11–14]. The available results are heterogeneous regarding perioperative complications and long-term outcome with 5-year survival ranging from 20 to 50% [13–15]. In addition, recent advances in liver surgery including the use of dynamic liver function tests e.g. LiMAx (maximum liver function capacity) or indocyanine green (ICG) and the increasing implementation of minimal-invasive liver surgery has broadened the disease spectrum in which LR appears feasible [16–21].

The aim of this study was to investigate short- and long-term outcomes in patients undergoing LR for rHCC compared to individuals with primary HCC in a monocentric European cohort of HCC patients.

## Material and methods

### Patients

The study comprised two hundred-twelve (*n* = 212) consecutive HCC patients who underwent curative-intent surgery at the University Hospital RWTH Aachen (UH-RWTH) between 2010 and 2020. Clinical staging was performed according to international guidelines and all individuals had localized tumors without signs of systemic disease. The study was conducted at the UH-RWTH in accordance with the requirements of the Institutional Review Board of the RWTH-Aachen University (EK 503/21), the current version of the Declaration of Helsinki, and the good clinical practice guidelines (ICH-GCP).

### Staging and surgical technique

All patients who were referred for surgical treatment to our institution underwent a detailed clinical work-up as previously described [2, 22]. Therefore, the number, size, and location of tumor nodules as well as the presence of distant metastases were evaluated by magnetic resonance imaging (MRI) or computed tomography (CT). The preoperative risk assessment was carried out based on the American society of anesthesiologists- (ASA) and the Eastern Cooperative Oncology Group (ECOG)-performance status, calculation of the future liver remnant (FLR) as well as parenchymal liver function as assessed by standard laboratory parameters and the LiMAx test (Humedics® GmbH, Berlin, Germany) [23]. Non-invasive liver function tests were routinely carried out, but no preoperative liver biopsies were obtained to assess the quality of the liver parenchyma. Patients staged Barcelona Clinic Liver Cancer (BCLC) A to C without any evidence of extrahepatic spread as well as compensated liver function were considered as candidates for surgery as primary treatment. The definitive decision for hepatectomy was made by a staff hepatobiliary surgeon and approved by the institutional interdisciplinary tumor board for every patient. If transplantation was suggested for the individual patient, the case was referred to and discussed within the local transplantation board. Transplantation was generally preferred over re-resection if age, comorbidities or contraindications e.g., active alcoholism as well as advanced tumor stages were not precluding this approach. In transplant candidates fulfilling the Milan criteria and therefore undergo exceptional model of end stage liver disease (exMELD) allocation, transplantation was considered as the primary treatment [24]. In patients with rHCC not fulfilling the Milan criteria who presented with compensated liver function, surgery was preferred as the primary treatment. In patients not undergoing exMELD allocation with severe liver dysfunction, the suggested treatment was determined within a case-by-case decision approach evaluating the chance of organ allocation by regular MELD allocation and a perioperative risk assessment. Liver resection was carried out in accordance with common clinical standards [2, 22]. In brief, an intraoperative ultrasound was performed to visualize the local tumor spread and other suspicious lesions. The decision for either anatomic resections or non-anatomic atypical wedge resections with an adequate resection margin was based on the surgeon’s preference. Parenchymal transection was carried out using the Cavitron Ultrasonic Surgical Aspirator (CUSA®, Integra LifeSciences®, Plainsboro NJ, USA) with low central venous pressure (CVP) and intermittent Pringle maneuvers if necessary in open hepatectomy. In laparoscopic hepatectomy, parenchymal transection was commonly performed by Thunderbeat ® (Olympus K.K., Tokyo, Japan), Harmonic Ace ® (Ethicon Inc. Somerville, NJ, USA) or laparoscopic CUSA (Integra life sciences, New Jersey, USA) in combination with vascular staplers (Echelon, Ethicon, Somerville, New Jersey, USA) or polymer clips (Teleflex Inc., Pennsylvania, USA). The anesthesiologic management was based on a restrictive fluid intervention strategy ensuring a low CVP during parenchymal dissection.

### Statistical analysis

The primary endpoint of this study was the statistical comparison between patients undergoing resection versus re-resection for HCC in terms of perioperative complications and long-term oncological outcome. Overall survival (OS) was defined as the period from surgery to the date of death from any cause or the last contact if the patient was alive. Recurrence-free survival (RFS) was measured from the date of resection to the date of first recurrence. RFS and OS in case of re-resection is defined from the time of re-resection until appearance of recurrence and the date of death from any cause or the last contact if the patient was alive, respectively. Patients who were free of tumor recurrence were censored at the time of death or at the last follow-up. Categorial data is presented in the form of numbers and percentages and compared using the chi-squared test, Fisher’s exact test or linear-by-linear association according to scale and number of cases. Data derived from continuous variables are presented as median and interquartile range and compared with the Mann–Whitney U-test. Survival curves were generated using Kaplan–Meier method and compared with the log-rank test. Median follow-up was assessed with the reverse Kaplan–Meier method. Complications are reported as in-hospital morbidity and mortality. Perioperatively deceased patients were included in all survival analyses. The level of significance was set to *p* < 0.05 and *p*-values are given for two-sided testing. Analyses were performed using SPSS Statistics 24 (IBM Corp., Armonk, NY, USA).

## Results

### Comparative analysis of the patient cohort

A total of 212 patients with a median age of 69 years who underwent curative-intent surgery for HCC at our institution from 2010 to 2020 were included in this study. Of these, a subgroup (*n* = 23) underwent re-resection due to rHCC and was compared to patients treated with surgery due to the primary HCC diagnosis (*n* = 189). More than half of the patients of both groups were male patients (resection group: 74.1% (140/189); re-resection group: 60.9% (14/23)) and assessed as ASA III or higher (resection group: 62.4% (118/189); re-resection group: 73.9% (17/23)). No differences between the groups regarding demographics and preoperative liver function despite a pronounced larger proportion of patients with non-alcoholic fatty liver disease (NAFLD) in the primary resection cohort (41.3% (78/189) vs. 8.7% (2/23), *p* = 0.014) were observed. With respect to preoperative imaging, a tendency for a lower median nodule count (1 vs. 2, *p* = 0.101) and significantly larger tumors (53 mm vs. 36 mm, *p* = 0.007) in the primary resection group was detected. No other examined imaging features e.g. macrovascular invasion (*p* = 0.372) were different between the groups. Of note, laparoscopic resections were much more common in the primary resection group (34.4% (65/189) vs. 4.3% (1/23), *p* = 0.003) whereas other operative features including intraoperative and postoperative transfusion characteristics displayed no significant difference. Perioperative complications were observed in more than half of the individuals of each group (resection group: 51.3% (97/189); re-resection group: 56.5% (13/23)) but showed a balanced distribution in the group-comparison analysis (*p* = 0.851). However, a lower rate of post hepatectomy liver failure (PHLF) according to the 50–50 criteria was observed in the resection group (1.1% (2/189) vs. 17.4% (4/23)) [21]. With respect to the pathological data, no difference in T category, microvascular invasion and tumor grading was found between the groups, but a tendency toward more R0 resections in the primary resection cohort (95.7% (180/189) vs. 87.0% (20/23) was displayed. The median time from initial treatment to re-resection in the rHCC cohort was 29 months (95% confidence interval (CI): 23–35 months). More clinicopathological and perioperative characteristics of both groups are outlined in Table 1 and a detailed overview about each case of the re-resection group is shown in Table 2.

**Table 1:** Comparative analysis of patients undergoing liver resection for hepatocellular carcinoma

Variables	Resection vs. Re-Resection analysis		
	Resection (*n* = 189)	Re-Resection (*n* = 23)	*p*-value
Demographics			
Gender, m/f (%)	140 (74.1)/49 (25.9)	14 (60.9)/9 (39.1)	.180
Age (years)	69 (60–75)	73 (62–78)	.204
BMI (kg/m^2^)	26 (23–29)	24 (21–28)	.160
Preoperative treatment			
Preoperative PVE, n (%)	8 (4.2)	2 (8.7)	.340
Preoperative TACE, n (%)	13 (6.9)	0	.194
Preoperative TARE, n (%)	3 (1.6)	0	.543
ASA, n (%)			.346
I	3 (1.6)	0	
II	68 (36.0)	6 (26.1)	
III	113 (59.8)	15 (65.2)	
IV	5 (2.6)	2 (8.7)	
V	0	0	
Liver disease, n (%)			**.014**
ALD	43 (22.8)	7 (30.4)	
NAFLD	78 (41.3)	2 (8.7)	
Viral	47 (24.9)	8 (34.8)	
Cryptogenic/others	21 (11.1)	6 (26.1)	
Preoperative liver function			
MELD Score	6 (6–7)	6 (6–7)	.758
AFP (ng/ml)	8 (3–51)	7 (4–11)	.574
Albumin (g/dl)	4.0 (3.6–4.4)	3.8 (3.6–4.3)	.179
AST (U/l)	41 (28–58)	35 (23–45)	.118
ALT (U/l)	33 (22–52)	32 (18–45)	.323
GGT (U/l)	100 (54–205)	80 (37–242)	.442
Total bilirubin (mg/dl)	0.5 (0.4–0.8)	0.7 (0.5–1.1)	.086
Platelet count (/nl)	216 (167–279)	211 (169–281)	.879
Alkaline phosphatase (U/l)	99 (75–139)	124 (97–161)	.018
Prothrombine time (%)	92 (84–100)	92 (84–100)	.930
INR	1.1 (1.0–1.1)	1.1 (1.0–1.1)	.788
Creatinine (mg/dl)	0.9 (0.7–1.1)	0.9 (0.7–1.0)	.650
Hemoglobin (g/dl)	13.3 (11.7–14.6)	13.0 (11.5–14.7)	.808
Child Pugh, n (%)			.962
A	172 (91.0)	21 (91.3)	
B	17 (9.0)	2 (8.7)	
Child Pugh score	5 (5–5)	5 (5–6)	.515
Preoperative imaging features			
Number of nodules	1 (1–2)	2 (1–3)	.101
Largest nodule diameter (mm)	53 (36–81)	36 (22–51)	**.007**
Tumor burden > 50%, n (%)	9 (4.8)	0	.285
Overall macrovascular invasion, n (%)	49 (25.9)	4 (17.4)	.372
Portal vein invasion, n (%)	32 (16.9)	3 (13.0)	.635
Extrahepatic vascular invasion, n (%)	12 (6.3)	1 (4.3)	.706
Portal vein thrombosis, n (%)	10 (5.3)	1 (4.3)	.847
Ascites, n (%)	8 (4.2)	0	.314
Operative data			
Laparoscopic resection, n (%)	65 (34.4)	1 (4.3)	**.003**
Conversation rate, n (%)	6 (9.2)	0	.750
Operative time (minutes)	204 (142–269)	240 (160–329)	.071
Operative procedure, n (%)			.166
Atypical	68 (36.0)	10 (43.5)	
Segmentectomy	27 (14.3)	1 (4.3)	
Bisegmentectomy	17 (9.0)	3 (13.0)	
Hemihepatectomy	46 (24.3)	2 (8.7)	
Extended liver resection	24 (12.7)	5 (21.7)	
ALPPS/TSH/other	7 (3.7)	2 (8.7)	
Additional procedures (RFA, etc.), n (%)	10 (5.3)	3 (13.0)	.143
Pringle maneuver, n (%)	11 (5.9)	3 (13.0)	.194
Duration of pringle maneuver (min)*	18 (11–21)	15 (5–24)	.692
Intraoperative blood transfusion, n (%)	53 (28.8)	8 (34.8)	.553
Intraoperative FFP, n (%)	73 (39.7)	11 (47.8)	.453
Intraoperative platelet transfusion, n (%)	4 (2.2)	1 (4.3)	.522
Pathological examination			
R0 resection, n (%)	180 (95.7)	20 (87.0)	.074
T category, n (%)			.133
T1	81 (43.1)	7 (33.3)	
T2	67 (35.46)	12 (57.1)	
T3/T4	40 (21.3)	2 (9.5)	
Microvascular invasion, n (%)	79 (45.9)	7 (30.4)	.160
Tumor grading, n (%)			.869
G1	13 (7.0)	1 (5.0)	
G2	139 (74.7)	16 (80.0)	
G3/G4	34 (18.3)	3 (15.0)	
Postoperative data			
Intensive care stay, days	1 (1–1)	1 (1–2)	.143
Hospitalization, days	8 (6–14)	8 (5–23)	.980
Postoperative complications, n (%)			.851
No complications	92 (48.7)	10 (43.5)	
Clavien-Dindo I	20 (10.6)	4 (17.4)	
Clavien-Dindo II	29 (15.3)	2 (8.7)	
Clavien-Dindo IIIa	19 (10.1)	2 (8.7)	
Clavien-Dindo IIIb	9 (4.8)	1 (4.3)	
Clavien-Dindo IVa	7 (3.7)	2 (8.7)	
Clavien-Dindo IVb	2 (1.1)	0	
Clavien-Dindo V	11 (5.8)	2 (8.7)	
PHLF 50–50 criteria*, n (%)	2 (1.1)	4 (17.4)	**.011**
PHLF ISGLS*, n (%)	39 (20.6)	4 (17.4)	.715
ISGLS Grade, n (%)			.179
A	26 (66.7)	1 (25.0)	
B	6 (15.4)	2 (50.0)	
C	7 (17.9)	1 (25.0)	
Postoperative blood transfusion	31 (16.8)	5 (21.7)	.560
Postoperative FFP	14 (7.6)	4 (17.4)	.116
Postoperative platelet transfusion	5 (2.7)	2 (8.7)	.135
Follow-up data			
Recurrence-free survival (months)	26 (17–35)	29 (2–56)	.675
Overall survival (months)	40 (31–49)	41 (33–49)	.612

Data presented as median and interquartile range if not noted otherwise. Follow-up data is presented as median and 95% CI. Categorical data were compared using the chi-squared test, Fisher’s exact test or linear-by-linear association according to scale and number of cases. Data derived from continuous variables of different groups were compared by Mann–Whitney U-Test. *Postoperative liver failure was assessed by the 50–50-criteria and the ISGLS definition [21, 44]

**Table 2:** Characteristics of patients undergoing re-resection for hepatocellular carcinoma

Patient number	Primary treatment				Recurrence treatment							
	Number of nodules	Largest diameter (mm)	Procedure	RFS (months)	Number of nodules	Largest diameter (mm)	Procedure	OR time (min)	Complications	Follow-up (months)	Alive (yes/no)	Recurrence (yes/no)
1	1	32	Hemihepatectomy	96	2	90	Bisementectomy + PVR	607	IVa	13	Yes	No
2	1	43	Segmentectomy	82	1	22	Extended hepatectomy	420	IIIb	64	Yes	Yes
3	1	24	Segmentectomy	67	1	16	Atypical	220	I	33	Yes	No
4	1	17	Segmentectomy	57	1	10	Atypical	150	I	41	No	No
5	1	24	Hemihepatectomy	46	1	25	Atypical	147	II	15	Yes	No
6	5	27	Atypical	42	3	40	Atypical	330	I	35	No	Yes
7	1	30	Atypical*	36	1	54	Bisegmentectomy	120	No	85	No	No
8	1	49	Atypical	33	1	31	Atypical	110	No	59	Yes	Yes
9	1	28	Atypical	32	2	51	Hemihepatectomy	229	IIIa	10	Yes	No
10	2	35	Atypical	31	1	7	Atypical	160	I	41	No	Yes
11	1	35	Atypical	29	4	49	Extended hepatectomy	240	II	26	Yes	No
12	1	36	Atypical	29	5	36	Extended hepatectomy	293	V	0	No	No
13	1	125	Hemihepatectomy	26	1	30	Atypical	269	No	6	Yes	No
14	2	15	Atypical*	25	4	25	Atypical	185	No	14	Yes	Yes
15	1	76	Bisegmentectomy	23	2	50	Bisegmentectomy	315	No	39	Yes	Yes
16	2	53	Bisegmentectomy	20	1	15	Atypical	70	No	17	Yes	No
17	1	60	Hemihepatectomy	16	1	130	Segmentectomy + PPPD	360	IVa	2	Yes	No
18	1	28	Segmentectomy	16	2	14	Hemihepatectomy	329	V	2	No	No
19	2	18	Atypical*	13	1	170	Bisegmentectomy	225	No	1	No	Yes
20	5	123	Hemihepatectomy	12	2	45	Atypical	180	No	18	No	n.a
21	1	53	Segmentectomy	11	3	80	Extended hepatectomy	240	No	71	Yes	No
22	1	52	Atypical	8	3	35	Extended hepatectomy	473	No	11	Yes	Yes
23	1	61	Hemihepatectomy	3	2	36	Atypical + HJ	300	IIIa	19	No	Yes

Detailed data of patients undergoing re-resection is shown. Complications were reported according to the Clavien-Dindo scale. *Cases were unsuccessfully pretreated by RFA or TACE/RFA prior initial liver resection

### Survival analysis

**Fig. 1:**
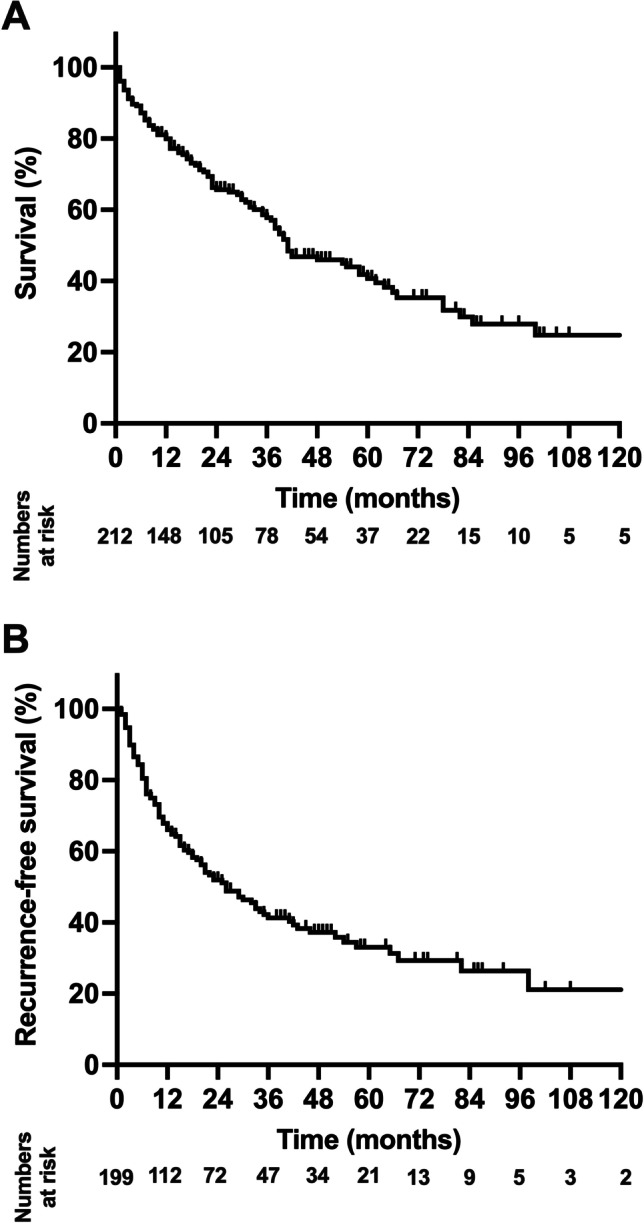
Oncological survival in hepatocellular carcinoma of the study cohort. **A**: Overall survival. The median OS of the cohort was 41 months (95%CI: 33–49 months). **B**: Recurrence-free survival. The median RFS of the cohort was 26 months (95%CI: 17–34 months). OS, overall survival; RFS, recurrence-free survival

**Fig. 2:**
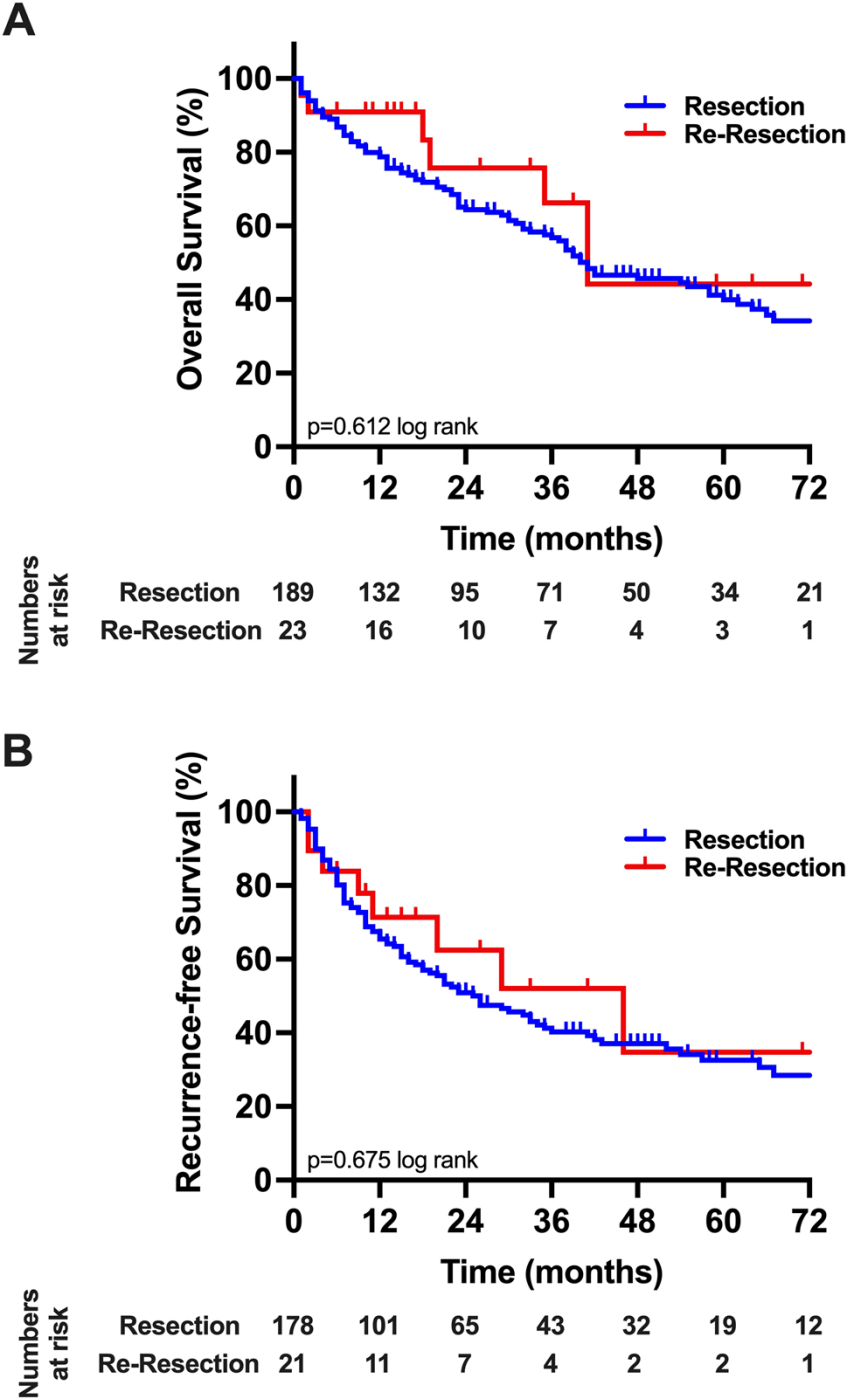
Oncological survival in hepatocellular carcinoma stratified by resection and re-resection. **A**: Overall survival. Patients undergoing primary resection showed a median OS of 40 months compared to 41 months in patients undergoing re-resection (*p* = 0.836 log rank). **B**: Recurrence-free survival. Patients undergoing primary resection showed a median RFS of 26 months compared to 29 months in patients undergoing re-resection (*p* = 0.946 log rank). OS, overall survival; RFS, recurrence-free survival

After a median follow-up of 50 months, the median OS of the cohort was 41 months (95%CI: 33–49 months; 3-year-OS = 58%, 5-year-OS = 41%) and the median RFS was 26 months (95%CI: 18–34 months; 3-year-RFS = 42%, 5-year-RFS = 33%; Fig. 1). Regarding the comparative analysis between the primary resection and re-resection groups, the median OS was 40 (95%CI: 31–49 months; 3-year-OS = 57%, 5-year-OS = 40%) in the resection cohort, while a median OS of 41 months (95%CI: 31–49 months; 3-year-OS = 66%, 5-year-OS = 44%) was observed in patients undergoing re-resection (*p* = 0.612 log rank; Fig. 2A). Also, no difference in RFS was detected between individuals undergoing primary resection (median RFS: 26 months (95%CI: 17–35 months), 3-year-RFS = 40%, 5-year-RFS = 33%) and patients treated by re-resection (median RFS: 29 months (95%CI: 2–56 months), 3-year-RFS = 52%,5-year-RFS = 35%; *p* = 0.675 log rank).

## Discussion

HCC represents one of the major global health issues with liver resection being the treatment of choice in patients with preserved liver function [1, 2, 8]. While the role of surgery in primary HCC is undoubtful supported with strong evidence, the ideal approach to rHCC is still under investigation and data focusing on Western patients are especially scarce [7, 10–14]. In a large cohort of European patients, we were able to demonstrate a notable short- and long-term outcome in patients with rHCC. Particularly, our data suggests that the oncological survival after LR for rHCC is similar to the survival after LR for primary HCC. Hence, LR should be considered as treatment option in European patients diagnosed with rHCC.

Suggested treatment modalities for rHCC range from re-resection and SLT over locoregional/interventional options or systematic therapies to best supportive care. The decision in favor of a certain therapy is based on the recurrence patterns, liver function, and overall physical performance status of the individual patient. Most experience regarding safety and oncological benefit of re-resection comes from Asian cohorts. In 1986, Nagasue et al. first reported a small series of 9 patients who underwent resection of rHCC [25]. Since then, multiple case-series from Eastern Asia have been published with the largest mono-centric cohort reported by Zou et al. comprising more than 600 patients [14]. In this study, the median OS after re-resection was 27 months which is notably shorter than in our admittedly smaller cohort of patients reported here (median OS 41 months). This is particularly interesting since multifocal disease (52.1% vs. 15.1%) as well as the tumors > 3 cm (60.9% vs. 13.0%) were more frequent in our cohort. However, microvascular invasion (30.4% vs. 55.7%) was less often detected which might at least in part explain the observed differences (Tables 1 and 2 [14]). The largest series on Western patients has been published by Roayaie et al. in 2010 and included 35 patients from Italy and the USA [26]. Interestingly, only patients with a single recurrent tumor on imaging and Child A liver disease were included which translates into an excellent median OS of 59 months (5-year-OS = 67%) in this study. More in line with our results is the report by Fabel et al. showing a median OS of 36 months (5-year-OS = 42%) in 31 patients treated by re-resection for rHCC [27]. While the survival data appears comparable between our study and the publication from Faber et al., our cohort displayed significantly higher morbidity (56.5% vs. 11.1%) and mortality (8.7% vs. 0%). However, it must be noted that our department policies also comprise surgery for patients staged BCLC A to C in both primary and recurrent HCC. Thus, our re-resection cohort also included complex cases treated by major liver resection and additional procedures such as portal vein resections (PVR) or the concomitant resection of adjacent organs (e.g. the pancreatic head (Table 2)).

While most of the available literature solely reports outcome in patients with rHCC, we additionally compared to short- and long-term outcome of patients undergoing LR for primary HCC. We noticed no statistical differences in a variety of pre-, intra- and postoperative characteristics except for larger proportion of NAFLD patients, more individuals undergoing laparoscopic resection and slightly larger tumors in patients with primary HCC resection. In contrast, posthepatectomy liver failure was more common in the rHCC cohort. Further, a statistically not significantly increased nodule count was observed in the re-resection cohort (Table 1). Overall, known oncological risk factors especially microvascular invasion and pathological staging were balanced between the resection and re-resection cohort, therefore allowing a meaningful comparative analysis regarding oncological outcome without further matching [28]. Particularly, we detected no difference in OS and RFS between patients undergoing resection or re-resection for HCC. This suggests that rHCC is per se not a prognostic factor for poor oncological outcome and indicates that these patients should not be treated differently from patients with the first diagnosis of HCC. As LR is universally recommended as a first-line treatment for primary HCC across common guidelines and expert opinions, re-resection should therefore also be considered in patients presenting with rHCC [7, 10].

Interestingly, in our study the rate of laparoscopic LR was notably higher in the resection than in the re-resection group. As laparoscopic LR was routinely implemented during the later study period, most patients undergoing re-resection were treated by open hepatectomy in the initial procedure. This and the complexity of the cases with rHCC as described above certainly explain the low rate of laparoscopic LR in the rHCC group. Even today, laparoscopic re-resection remains challenging in case of rHCC due to the formation of intraabdominal adhesions. Currently, only a few studies are available focusing on laparoscopic re-resection in rHCC [29–32]. However, a recent meta-analysis based on Eastern patients showed that laparoscopic re-resection for rHCC is associated with fewer overall complication and a shorter hospitalization but displayed similar 90-day mortality compared to conventional re-resection [33].

While large studies from Asia regarding the role of re-resection for rHCC already exist, it is yet to be explored whether these results are unconditionally transferable to Western patients [9]. The general approach to HCC seems to be more aggressive in Asian countries. This might partially be explained by the larger proportion of viral etiology in Eastern patients which results in a generally younger HCC population with often less severe underlying cirrhosis and fewer comorbidities [34]. Therefore, adjusted staging system, e.g. Hong Kong Liver Cancer staging (HKLC), Japanese Integrated System (JIS) or Chinese University Integrated System (CUPI), are used to guide treatment in Asian patients but are less useful in Western populations [35, 36]. Even genomic characteristics vary between Asian and European patients [37]. Thus, treatment recommendation for HCC in European patients, especially in the complex situation of rHCC, should preferably be based on data from European cohorts.

Our long-term oncological results are at the price of significant morbidity and mortality. This gives rise to the question of alternative treatment modalities. SLT is often proposed as it resolves the issue of underlying liver disease and treats potential micrometastases. However, the procedure itself is also associated with a relatively high morbidity [38]. Notably, a recent meta-analysis comparing SLT to other treatment modalities confirmed the advantage of SLT in long-term outcomes [39]. In addition, the direct comparison of re-resection with SLT within a subgroup analysis showed preferable 3- and 5-year-RFS for the SLT cohort [39]. Nonetheless, especially in Germany with one of the lowest number of available deceased donor allografts among Western countries, the use of SLT is strongly limited by the shortage of available donor grafts. Radiofrequency ablation (RFA) is also suggested to be a valid alternative for re-resection. In particular, RFA is nowadays considered to be equivalent to LR in small solitary HCC and, therefore, recommended by various guidelines [7, 10, 40]. However, data basis for rHCC is yet to be fully unraveled. In a recent monocentric report from Singapore, re-resection was associated with a late survival benefit but displayed a higher procedural morbidity rate [41]. In contrast, combined data from China and Italy displayed longer RFS upon re-resection but failed to convey an improved OS compared to RFA [42]. Similar to the report by Chua et al., morbidity was also higher after re-resection in this study [41]. While therapeutic alternatives for re-resection are certainly clinically appealing, our analysis cannot enlighten this issue, and further investigations in a randomized setting are required to determine the ideal approach to rHCC.

Whether time to recurrence from primary resection should guide treatment decisions is currently debated within the international literature [28, 43]. Our rHCC cohort comprised patients with disease recurrence after less than 1 year up to patients with a DFS of more than 5 years underlining our surgical approach in every feasible recurrence situation. To further investigate this issue of early versus late recurrence, we pragmatically split our rHCC subgroup into two almost equal sized groups (cut-off 26 months) and observed a tendency for longer OS and DFS in patients experiencing later recurrence (supplementary Figure S1, supplementary Table S1). While our dataset does not allow to elaborate further statistically associations here, this observation is in line with previous reports investigating the late versus early recurrence topic [28, 43]. Of note, in a recent publication of Wei et al., individuals undergoing curative treatment approach for late recurrence displayed comparable OS with patients without disease relapse and patients with early recurrence showed inferior OS after curative re-treatment compared to patients with no recurrence, yet still a better outcome than patients undergoing palliative treatment for early recurrence which underlines the role of surgery in both scenarios [43].

Like any other observational clinical study, our analysis has certain inherent limitations. All HCC patients analyzed in this study underwent treatment in a monocentric setting reflecting our individual clinical approach to this disease and the study is based on a retrospective data collection which was not obtained in the setting of a controlled clinical trial. This also results in large proportion of ASA III patients and individuals with higher BCLC stages due to our aggressive treatment approach. Moreover, our data set appears small compared to some other studies especially from Asian cohorts. Further, our relatively small data set did not allow to conduct a meaningful sub-analysis investigating clinical and oncological differences between early and late disease recurrences.

## Conclusion

Notwithstanding the aforementioned limitations, we show that re-resection is technically feasible and safe in patients with rHCC. Further, comparative analysis displayed similar oncological outcome in patients with primary and rHCC treated by liver resection. Re-resection should therefore be considered in European patients diagnosed with rHCC.

## Supplementary Information

Below is the link to the electronic supplementary material.

Supplementary file1 (DOCX 431 KB)

Supplementary file2 (DOCX 71 KB)

## Abbreviations

ALD: Alcoholic liver disease
ALT: Alanine aminotransferase
ALPPS: Associating liver partition with portal vein ligation for staged hepatectomy
AP: Alkaline phosphatase
ASA: American society of anesthesiologists
AST: Aspartate aminotransferase
BCLC: Barcelona Clinic Liver Cancer
BMI: Body mass index
CPS: Child Pugh Score
CI: Confidence interval
CRP: C-reactive protein
CT: Computed tomography
CUPI: Chinese University Integrated System
CUSA: Cavitron Ultrasonic Surgical Aspirator
CVP: Central venous pressure
ECOG: Eastern Cooperative Oncology Group
FFP: Fresh frozen plasma
FLR: Future liver remnant
GCP: Good clinical practice
GGT: Gamma glutamyltransferase
HCC: Hepatocellular carcinoma
HJ: Hepaticojejunostomy
HKLC: Hong Kong Liver Cancer
ICG: Indocyanine green
INR: International normalized ratio
ISGLS: International Study Group of Liver Surgery
JIS: Japanese Integrated System
LiMAX: Maximum liver function capacity
LR: Liver resection
MELD: Model of end-stage liver disease
MRI: Magnetic resonance imaging
NAFLD: Non-alcoholic fatty liver disease
OS: Overall survival
PHLF: Posthepatectomy liver failure
PVE: Portal vein embolization
PVR: Portal vein resection
RFA: Radiofrequency ablation
RFS: Recurrence-free survival
rHCC: Recurrent HCC
SLT: Salvage liver transplantation
TACE: Transarterial chemoembolization
TARE: Transarterial radioembolization
TSH: Two-stage hepatectomy
UH-RWTH: University Hospital Rheinisch-Westfälische Technische Hochschule
UICC: Union for international cancer control
