# Supplementary material for: The role of re-resection in recurrent hepatocellular carcinoma
Source: Langenbecks Arch Surg. 2022 May 23;407(6):2381–91. doi: 10.1007/s00423-022-02545-1 (PMC9468093; doi:10.1007/s00423-022-02545-1)
Supplement: Supplementary file 1 — Supplementary file1 (DOCX 431 KB) [file 423_2022_2545_MOESM1_ESM.docx]

**Supplementary Figure 1: Oncological survival in recurrent hepatocellular carcinoma stratified by time to recurrence**

**
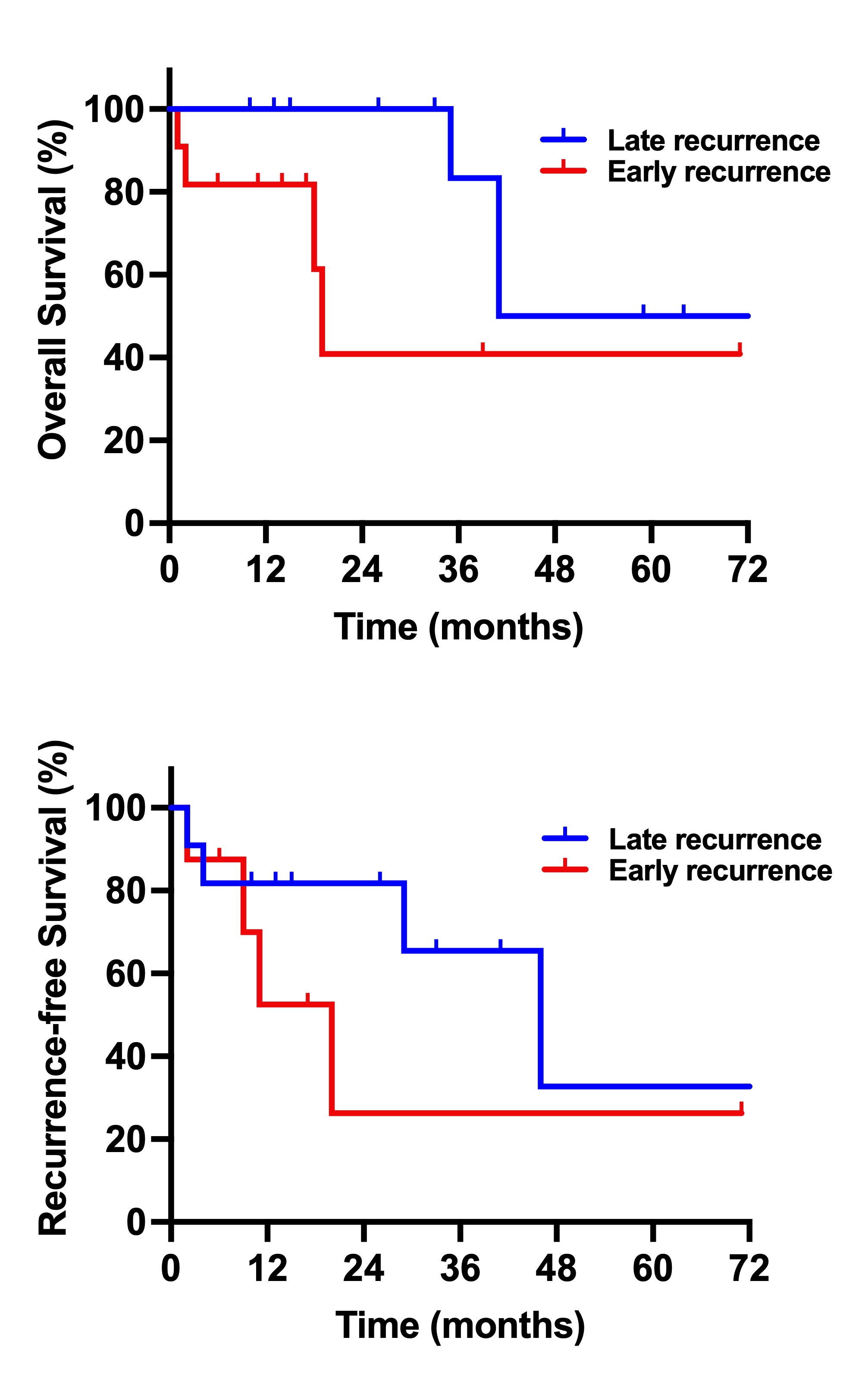
**

Patients undergoing surgery for recurrent HCC were stratified by time to recurrence after initial resection using the cut-off of 26 months (early vs. late recurrence) to create equal sized subcohorts. The statistical validity is impaired by small sample size in both groups. The overall survival was months 19 in the early recurrence group while 41 months in the late recurrence group. Correspondingly, the recurrence-free survival was 11 months in the early recurrence group and 46 months in the late recurrence group.
