# Supplementary material for: The role of re-resection in recurrent hepatocellular carcinoma
Source: Langenbecks Arch Surg. 2022 May 23;407(6):2381–91. doi: 10.1007/s00423-022-02545-1 (PMC9468093; doi:10.1007/s00423-022-02545-1)
Supplement: Supplementary file 2 — Supplementary file2 (DOCX 71 KB) [file 423_2022_2545_MOESM2_ESM.docx]

**Supplementary Table 1: Comparative analysis of patients undergoing liver resection for hepatocellular carcinoma with respect to early versus late recurrence**

| **Variables** |  | **Late recurrence (n=12)** | **Early recurrence (n=11)** |
| --- | --- | --- | --- |
| **Demographics** |  |  |  |
| Gender, m/f |  | 9/3 | 5/6 |
| Age (years) |  | 74 (70 – 79) | 65 (54 – 77) |
| BMI (kg/m^2^) |  | 24 (20 – 28) | 24 (22 – 29) |
| Preoperative treatment |  |  |  |
| Preoperative PVE, n |  | 1 | 1 |
| Preoperative TACE, n |  | 0 | 0 |
| Preoperative TARE, |  | 0 | 0 |
| ASA, n |  |  |  |
| I |  | 0 | 0 |
| II |  | 1 | 5 |
| III |  | 9 | 6 |
| IV |  | 2 | 0 |
| V |  | 0 | 0 |
| Liver disease, n |  |  |  |
| ALD |  | 6 | 1 |
| NAFLD |  | 0 | 2 |
| Viral |  | 4 | 4 |
| Cryptogenic/others |  | 2 | 4 |
|  |  |  |  |
| **Preoperative liver function** |  |  |  |
| MELD Score |  | 6 (6 – 8) | 6 (6 – 6) |
| AFP (ng/ml) |  | 4 (2 – 8) | 11 (6 – 2586) |
| Albumin (g/dl) |  | 3.8 (3.4 – 4.4) | 3.8 (3.7 – 4.3) |
| AST (U/l) |  | 34 (23 – 43) | 38 (23 – 58) |
| ALT (U/l) |  | 29 (15 – 55) | 32 (24 – 40) |
| GGT (U/l) |  | 69 (36 – 439) | 83 (37 – 242) |
| Total bilirubin (mg/dl) |  | 0.7 (0.4 – 1.2) | 0.7 (0.5 – 1.12) |
| Platelet count (/nl) |  | 244 (152 – 307) | 178 (169 – 262) |
| Alkaline Phosphatase (U/l) |  | 133 (96 – 460) | 118 (102 – 140) |
| Prothrombine time (%) |  | 92 (80 – 95) | 98 (85 – 106) |
| INR |  | 1.1 (1.0 – 1.2) | 1.0 (1.0 – 1.1) |
| Creatinine (mg/dl) |  | 0.9 (0.8 – 1.1) | 0.7 (0.6 – 0.9) |
| Haemoglobin (g/dl) |  | 12.5 (10.6 – 14.9) | 13.2 (11.9 – 14.7) |
| Child Pugh, n |  |  |  |
| A |  | 10 | 11 |
| B |  | 2 | 0 |
| Child Pugh score |  | 5 (5 – 6) | 5 (5 – 5) |
|  |  |  |  |
| **Preoperative Imaging features** |  |  |  |
| Number of nodules |  | 1 (1 – 3) | 2 (1 – 3) |
| Largest nodule diameter (mm) |  | 34 (18 – 51) | 36 (25 – 80) |
| Tumor burden > 50%, n |  | 0 | 0 |
| Overall macrovascular invasion, n |  | 3 | 1 |
| Portal vein invasion, n |  | 2 | 1 |
| Extrahepatic vascular invasion, n |  | 1 | 0 |
| Portal vein thrombosis, n |  | 1 | 0 |
| Ascites, n |  | 0 | 0 |
|  |  |  |  |
| **Operative Data** |  |  |  |
| Laparoscopic resection, n |  | 0 | 1 |
| Conversation rate, n |  | 0 | 0 |
| Operative time (minutes) |  | 225 (148 – 321) | 269 (185 – 329) |
| Operative procedure, n |  |  |  |
| Atypical |  | 6 | 4 |
| Segmentectomy |  | 0 | 1 |
| Bisegmentectomy |  | 1 | 2 |
| Hemihepatectomy |  | 1 | 1 |
| Extended liver resection |  | 3 | 2 |
| ALPPS/TSH/other |  | 1 | 1 |
| Additional procedures (RFA, etc.), n |  | 1 | 2 |
| Pringle maneuver, n |  | 2 | 1 |
| Duration of pringle maneuver (min)* |  | 5/10 | 24 |
| Intraoperative blood transfusion, n |  | 6 | 2 |
| Intraoperative FFP, n |  | 8 | 3 |
| Intraoperative platelet transfusion, n |  | 1 | 0 |
|  |  |  |  |
| **Pathological examination** |  |  |  |
| R0 resection, n |  | 11 | 9 |
| T category, n |  |  |  |
| T1 |  | 5 | 2 |
| T2 |  | 5 | 7 |
| T3/T4 |  | 1 | 1 |
| Microvascular invasion, n |  | 4 | 3 |
| Tumor grading, n |  |  |  |
| G1 |  | 1 | 0 |
| G2 |  | 8 | 8 |
| G3/G4 |  | 1 | 2 |
|  |  |  |  |
| **Postoperative Data** |  |  |  |
| Intensive care stay, days |  | 1 (1 – 3) | 1 (1 – 1) |
| Hospitalization, days |  | 10 (6 – 22) | 7 (5 – 34) |
| Postoperative complications, n |  |  |  |
| No complications |  | 2 | 8 |
| Clavien-Dindo I |  | 4 | 0 |
| Clavien-Dindo II |  | 2 | 0 |
| Clavien-Dindo IIIa |  | 1 | 1 |
| Clavien-Dindo IIIb |  | 1 | 0 |
| Clavien-Dindo IVa |  | 1 | 1 |
| Clavien-Dindo IVb |  | 0 | 0 |
| Clavien-Dindo V |  | 1 | 1 |
| PHLF 50-50 criteria*, n |  | 1 | 1 |
| PHLF ISGLS*, n |  | 3 | 1 |
| ISGLS Grade, n |  |  |  |
| A |  | 1 | 0 |
| B |  | 1 | 1 |
| C |  | 1 | 0 |
| Postoperative blood transfusion, n |  | 2 | 1 |
| Postoperative FFP, n |  | 4 | 2 |
| Postoperative platelet transfusion, n |  | 0 | 0 |
|  |  |  |  |
| **Follow-up Data** |  |  |  |
| Recurrence-free survival (months) |  | 46 (21 – 71) | 11 (0 – 22) |
| Overall survival (months) |  | 41 (16 – 66) | 19 (17 – 21) |

Data presented as median and interquartile range if not noted otherwise. Follow-up data is presented as median and 95% CI. Categorical data were compared using the chi-squared test, fisher’s exact test or linear-by-linear association according to scale and number of cases. Data derived from continuous variables of different groups were compared by Mann-Whitney-U-Test. *Postoperative liver failure was assessed by the 50-50-criteria and the ISGLS definition [1,2] . ALD, alcoholic liver disease; ALPPS; Associating liver partition with portal vein ligation for staged hepatectomy; ALT, alanine aminotransferase; ASA, American society of anesthesiologists classification; AST, aspartate aminotransferase; BCLC, Barcelona clinical liver cancer staging system; BMI, body mass index; CI, confidence interval. FFP, fresh frozen plasma; GGT, gamma glutamyltransferase; INR, international normalized ratio; ISGLS, International Study Group of Liver Surgery; MELD, model of end stage liver disease; NAFLD, Non-alcoholic fatty liver disease; PHLF, Posthepatectomy liver failure; PVE; portal vein embolization; RFA, radiofrequency ablation; TACE, transarterial chemoembolization; TARE, transarterial radioembolization; TSH, Two-stage hepatectomy.

**References**

1. Balzan S, Belghiti J, Farges O, Ogata S, Sauvanet A, Delefosse D, Durand F (2005) The "50-50 criteria" on postoperative day 5: an accurate predictor of liver failure and death after hepatectomy. Annals of surgery 242 (6):824-828, discussion 828-829

2. Rahbari NN, Garden OJ, Padbury R, Brooke-Smith M, Crawford M, Adam R, Koch M, Makuuchi M, Dematteo RP, Christophi C, Banting S, Usatoff V, Nagino M, Maddern G, Hugh TJ, Vauthey JN, Greig P, Rees M, Yokoyama Y, Fan ST, Nimura Y, Figueras J, Capussotti L, Buchler MW, Weitz J (2011) Posthepatectomy liver failure: a definition and grading by the International Study Group of Liver Surgery (ISGLS). Surgery 149 (5):713-724. doi:10.1016/j.surg.2010.10.001
